# circDENND4C Promotes Proliferation and Metastasis of Lung Cancer by Upregulating BRD4 Signaling Pathway

**DOI:** 10.1155/2021/2469691

**Published:** 2021-11-28

**Authors:** Dongjie Ma, Yingzhi Qin, Shanqing Li, Li Li, Jia He, Yeye Chen, Xiaoyun Zhou, Hongsheng Liu

**Affiliations:** Department of Thoracic Surgery, Peking Union Medical College Hospital, Peking Union Medical College and Chinese Academy of Medical Sciences, Beijing 100730, China

## Abstract

**Objective:**

To investigate the effects of circDENND4C on the malignant biological behavior of lung cancer and its downstream target genes and molecular mechanisms.

**Methods:**

The expression of circDENND4C in lung cancer tissues and cells was detected. After transfection with silenced circDENND4C, the expression levels of circDENND4C, miR-141-3p, and BRD4 in lung cancer cells were detected by qRT-PCR. The targeting relationship between circDENND4C and miR-141-3p as well as miR-141-3p and BRD4 was verified. Cell activity was detected by CCK-8 and EdU assay. Transwell assay was used to detect the invasiveness of A549 and NCI-H1299 in each group. Effects of circDENND4C on proliferation and metastasis of lung cancer in nude mice were studied.

**Results:**

In vitro and in vivo results showed that circDENND4C silencing reduced the proliferation, invasion, and metastasis of lung cancer cells. Mechanism studies showed that circDENND4C has a targeting relationship with miR-141-3p. However, miR-141-3p has a targeting relationship with BRD4. circDENND4C indirectly upregulated BRD4 through sponge adsorption of miR-141-3p, thereby promoting metastasis and proliferation of NSCLC.

**Conclusion:**

circDENND4C, as an oncogene, promotes the proliferation, invasion, and metastasis of lung cancer cells.

## 1. Introduction

With the development of social economy and environmental pollution, the incidence of various cancers and lung-related diseases (such as lung cancer, pneumonia, *tuberculosis*, and hypoxic pulmonary hypertension) has increased significantly [1]. Pulmonary diseases are difficult to treat, which is why finding effective prevention and detection methods is the focus of research [2–5].

circRNA is widely expressed in organisms and plays an important role in regulating biological functions [6]. circRNA is highly specific, stable, and conservative and has great potential in the diagnosis and treatment of diseases [7, 8]. The role of circRNA in lung cancer has been extensively studied, but its role as a biomarker and its mechanism of action still need to be further elucidated [9, 10]. In breast cancer, circDENND4C silencing can inhibit glycolysis, migration, and invasion of breast cancer under hypoxia [11]. In addition, circDENND4C promotes breast cancer cell proliferation under hypoxia [12]. circDENND4C also promotes colorectal cancer cell proliferation, migration, and glycolysis [13]. However, the role of circDENND4C in lung cancer has not been reported.

miRNAs are related to tumor invasion, metastasis, and drug resistance [14, 15]. miRNAs can be used as biomarkers for early screening, diagnosis, and prognostic evaluation [16]. miR-141-3p has low expression in breast cancer tissues and cells [17]. Increased miR-141-3p expression inhibited epithelial-mesenchymal transformation in breast cancer cells [17]. However, the functions of miR-141-3p on lung cancer cells need to be further studied. As a member of the Bromodomain and extraterminal domain (BET) family, BRD4 plays a pivotal role in the development of liver cancer, leukemia, breast cancer, pancreatic cancer, and malignant melanoma [18–21]. However, the role of BRD4 in lung cancer needs further research.

This study analyzed the expression of circDENND4C in non-small cell lung cancer (NSCLC) tissues and cells. The target genes were predicted and verified by bioinformatics analysis. To study the effect of circDENND4C on the expression levels of miR-141-3p and BRD4, the effects of circDENND4C on proliferation, invasion, and metastasis of NSCLC cells were analyzed by in vivo and in vitro experiments.

## 2. Methods

### 2.1. Lung Cancer Tissue and Case Collection

Twenty NSCLC patients who admitted to the oncology department of our hospital from November 2020 to February 2021 were selected. All patients were resected and confirmed by case diagnosis. The patients included 11 males and 9 females whose ages ranged from 46 to 78 years, with a median age of 67.1 ± 9.4 years. Tumors were graded according to UICC TNM standard 7. All patients underwent radical surgery without chemotherapy, and all patients signed informed consent. All specimens were identified by two specialists with the title of deputy senior physician or above in the pathology department of our hospital. The study was approved by the hospital Ethics Committee of Peking Union Medical College.

### 2.2. Cell Culture

Lung cancer cells A549, H1299, H446, H460, and H1792 and normal human lung epithelial cells BEAS-2B were purchased from American Type Culture Collection (ATCC, Manassas, VA, USA) and cultured in RPMI 1640 medium (10% FBS) (Gibco, Life Technologies, Rockville, MD, USA). They were then placed in a 37°C 5% CO_2_ incubator (Thermo Fisher Scientific, Waltham, MA, USA). When the cells melted to about 80%, trypsin was added for digestion and passage. Logarithmic growth phase cells were taken for experiment.

### 2.3. Cell Transfection

Transfection was then performed. Lipofectamine™ 2000 (Life Technologies, Rockville, MD, USA) was used to transfect miR-con, miR-141-3p mimics, inhibitor-NC, miR-141-3p inhibitor, si-NC, and si-BRD4 into cells. The overexpressed plasmid uses pcDNA 3.1 [22, 23]. Plasmids are constructed and purchased from GenePharma (Shanghai, China). Cells without any treatment were used as blank control CCK-8 to collect single cell suspension digested by transfected cells in each group. The cell density was adjusted to 5 × 104/mL. Then, 100 *μ*L cell suspension was placed in the culture well, and the blank control group was set. Three multiple wells were set in each group. After 72 hours of preculture, fresh medium was replaced every 1 day. Afterward, 10 *μ*L CCK-8 solution (Solarbio, Beijing, China) was added to each well and then incubated for 2 h in the incubator. The optical density (*D*) value at 450 nm was detected under a microplate analyzer, and the multiplication ratio was calculated as follows: (*D* experiment-d blank)/(*D* control-d blank). The experiment was repeated three times.

### 2.4. EdU

Logarithmic growth phase cells were collected and inoculated into 24-well plates with about 5 × 10^4^ cells per well. They were then incubated in a constant temperature pack for 24 h. EdU solution (Beyotime, Shanghai, China) was diluted at 2500 : 1 in complete cell culture medium. Then, 200 *μ*L and 50 *μ*mol/L EdU medium were added to each well and incubated for 2 h. This step was followed by the addition of 200 *μ*L cell fixative solution (4% paraformaldehyde) to each well and 30 min incubation at room temperature. Afterward, 2 mg/mL glycine (prepared by ddH_2_O) was added to each well and incubated in a decolorization shaker for 5 min. Decolorization shaker was cleaned for 5 min. Then, 200 *μ*L penetrant (0.5% Triton X-100 PBS) was added to each well and incubated in a decolorization shaker for 10 min. Next, the sample was washed with PBS once for 5 min, 200 *μ*L of 1 × Apollo dyeing reaction was added to each well (prepared according to the instructions), and then it was incubated in a decolorizing shaking bed at room temperature away from light for 30 min. This step was followed by the addition of 200 *μ*L methanol to each well and two times of cleaning. DAPI staining was conducted by adding 200 *μ*L reaction solution to each well, hiding the sample from light, and incubating it for 5 min at room temperature. After staining, observations were conducted immediately and images were collected. EdU-positive cells were counted by ImageJ (V 1.8.0).

### 2.5. Fluorescence In Situ Hybridization Experiment

The RNA-FISH hybridization kit of RiboBio (Guangzhou, China) was used for the experiment according to the instructions. The cells were fixed in 4% paraformaldehyde for 24 h. PBS buffer of 0.1% Triton X-100 was used for permeation at 4°C for 8 min. Prehybridization solution was added at 37°C for 30 min. Fluorescence labeled hybridization probe and buffer were added overnight at 37°C. Then, 42°C citric acid buffer was used for washing, and DAPI was added for nucleation for 8 min. Laser confocal microscope (Nikon, Japan) was used for image acquisition.

### 2.6. Transwell Experiment

The cell concentration was adjusted to 2 × 10^4^ cells/mL. Then, 200 *μ*L cell suspensions were, respectively, inoculated into the upper chamber of Matrigel coated (BD Bioscience, USA) Transwell chamber (Millipore, Billerica, MA, USA). Culture medium was added to the lower chamber. After 24 h, the cells were fixed in 4% paraformaldehyde and then stained with 0.1% crystal violet. Five fields were randomly selected.

### 2.7. Clone Formation Experiment

The cells were digested 48 h after transfection. The cell concentration was added to 500/mL. Then, the cells were inoculated in 6-well plates, with 2 mL in each well. The culture medium was changed at two-day intervals. The growth of cell colonies was observed after 14 days of culture. Culture was terminated when colony formation was visible to the naked eye. Colonies of more than 50 cells were counted as 1 clone. Clone formation rate = number of cloned cells formed/number of inoculated cells × 100%. This process was repeated three times.

### 2.8. qRT-PCR

After culturing for 48 hours, the cells were collected and lysed with TRIzol reagent, and then the total RNA was extracted. Then, 1 *μ*g of the sample reverse transcribed into cDNA was taken. Next, 2 *μ*l of the reverse transcription sample was taken, corresponding primers were added, and SYBR Premix EX Taq II kit was used (TaKaRa Biotechnology Co., Ltd., Dalian, China) for amplification reaction. PCR was conducted according to the instructions of the fluorescence quantification kit. The reaction conditions are as follows: 50°C for 2 minutes, 95°C for 5 minutes, 95°C for 30 s, 55°C for 30 s, and 72°C for 30 s, with 40 cycles in total. Dissolution curve analysis was conducted at 60°C for 30 min. The experiment was repeated three times. The sequence is as follows: BRD4, F: 5′-GCACAATCAAGTCTAAACTGGAG-3′, R: 5′-TCATGGTCAGGAGGGTTGTAC-3′; GAPDH, F: 5′-GGCTGAGAACGGGAAGCTTGTCAT-3′, R: 5′-CAGCCTTCTCCATGGTGGTGAAGA-3′. The relative expression is calculated by the 2^−△△Ct^ method [24].

### 2.9. Subcutaneous Tumor-Bearing Model

Twelve four-week-old male nude mice were randomly divided into two groups, with six mice in each group, and fed in cages. The rearing environment is a barrier system. Mice were fed in SPF environment. The drinking water of nude mice was purified water with high-pressure sterilization. The mice were subjected to adaptive feeding for one week. When the cells grew to 80%, they were digested and centrifuged at 1000*g*/min for 5 min. The cells were suspended at 2 × 10^6^ cells/0.2 mL. The tumor model was established by inoculating the cells subcutaneously into the back of the right hind limb of the mice. The weight of the mice was measured and recorded. Five weeks later, the nude mice were killed by euthanasia and the tumor tissue was immediately exfoliated. Tumor volume was recorded every 2 days during the experiment. A denotes the mean long diameter, and B denotes the mean short diameter; tumor volume = 1/2 × *A* × *B*^2^. All animal experiments were approved by the animal Ethics Committee of Peking Union Medical College.

### 2.10. H&E Dye

Nude mouse lung tissue was removed and fixed in 4% paraformaldehyde solution at room temperature for 48 h. It was then dehydrated, paraffin-embedded, and sectioned. Sections were stained with H&E dye. Changes in lung metastases were observed under a microscope.

### 2.11. TargetScan and Starbase Site Analysis

TargetScan (http://www.targetscan.org/vert_71/) database was used to predict the miRNA target genes. The StarBase (http://starbase.sysu.edu.cn/) website was used to predict circRNA and the combination of microRNAs.

### 2.12. Double Luciferase Reporter Gene Assay

circDENND4C and BRD4 sequences containing miR-141-3p binding sites were amplified by PCR and then constructed into a luciferase expression vector. circDENND4C and BRD4 wild-type vectors (WT) and mutated vectors (MUT) were obtained. WT-BRD4 and MUT-BRD4 were cotransfected into cells with miR-con and miR-141-3p mimics by using the liposome method. The medium was discarded 36 h after total transformation and washing with PBS three times. Lysate was added and then the sample was shaken for 30 min until the cells were completely lysed. LARII was added to detect the luciferase activity of firefly and sea kidney, and the luciferase/kidney luciferase ratio was calculated. The experiment was repeated three times.

### 2.13. Statistical Analysis

SPSS 20.0 (SPSS Inc., Chicago, IL, USA) statistical software and GraphPad Prism 7 (Media Cybernetics, Silver Spring, MD, USA) were used to draw relevant pictures of the experimental data. Measurement data were expressed as mean ± standard deviation. Unpaired *t*-test was used for comparison between the two groups. A comparison of the mean values between multiple groups was analyzed by ANOVA. *p* < 0.05 or *p* < 0.01 indicated that the difference was statistically significant.

## 3. Results

### 3.1. circDENND4C Expression in Lung Cancer Tissues, Lung Cancer Cells, and Normal Lung Cells

**Figure 1:**
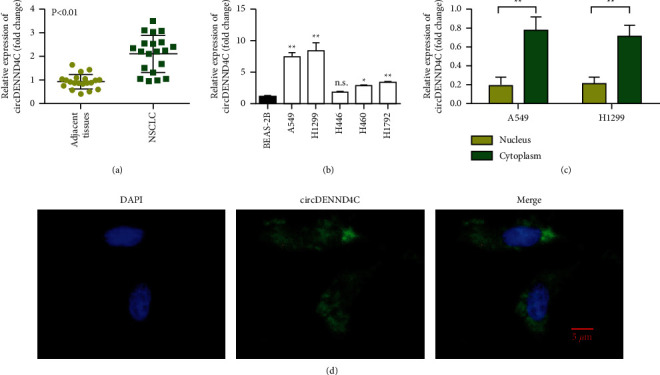
circDENND4C is upregulated in NSCLC. (a) RT-qPCR analysis of circDENND4C expression in NSCLC tissues. (b) Differential expression pattern of circDENND4C in NSCLC cell line. circDENND4C is highly expressed in A549 and H1299 cell lines. (c) The relative expression of circDENND4C in the nucleus and cytoplasm. The results showed that circDENND4C was mainly expressed in the cytoplasm. (d) RNA-FISH localizes circDENND4C in the cytoplasm of NSCLC. ^∗^*P* < 0.05 and ^∗∗^*P* < 0.01.

The expression level of circDENND4C in lung cancer tissues was significantly increased compared with that in normal adjacent lung tissues (Figure 1(a), *p* < 0.01), and circDENND4C in lung cancer cells A549, H1299, H460, and H1792 was also increased compared with normal lung cells BEAS-2B (Figure 1(b)). Further results showed that circDENND4C mainly distributed in cytoplasm (Figures 1(c) and 1(d)).

### 3.2. Effects of Silencing circDENND4C on A549 and H1299 Cells

**Figure 2:**
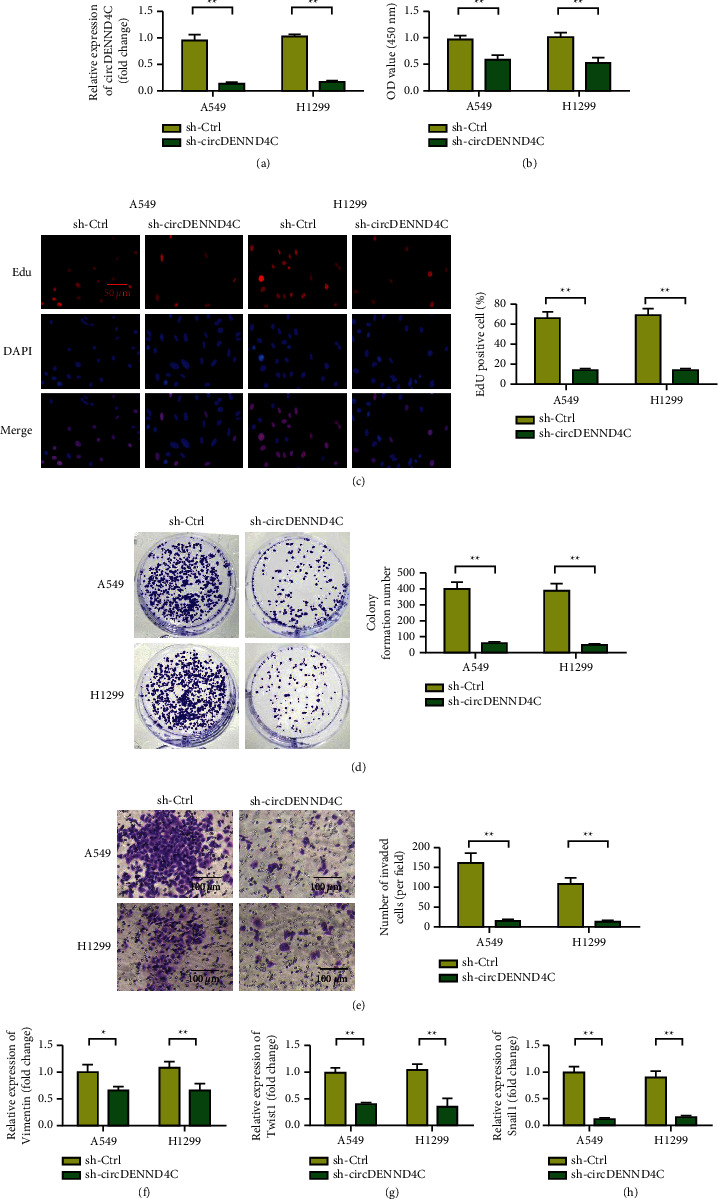
Downregulation of circDENND4C inhibits the proliferation and invasion of NSCLC cells in vitro. (a) The qRT-PCR method was used to detect the transfection effect of NSCLC cell lines (A549 and H1299). (b) The CCK-8 method was used to detect the cell viability and proliferation of H1299 and A549 cells in the circDENND4C downregulation group (sh-circDENND4C) and the negative control group (sh-ctrl). (c) The EdU method was used to detect the proliferation ability of H1299 and A549. (d) The colony formation test was used to detect the colony formation ability of the cells in the downregulated circDENND4C group compared with the sh-ctrl group. (e) Transwell experiment measures the changes in migration ability of NSCLC cell lines after different treatments. (f) PCR was used to detect the expression of Vimentin in sh-circDENND4C and sh-Ctrl groups. (g) PCR was used to detect the expression of Twist1 in sh-circDENND4C and sh-Ctrl groups. (h) PCR was used to detect the expression of Snail1 in the sh-circDENND4C and sh-Ctrl groups ^∗^*P* < 0.05 and ^∗∗^*P* < 0.01.

qRT-PCR results showed that the expression level of circDENND4C in the sh-circDENND4C group was significantly reduced compared with that in the blank control group (Figure 2(a), *p* < 0.01). The CCK-8 method showed that, compared with the blank control group, the activity of lung cancer cells in the sh-circDENND4C group was significantly decreased (Figure 2(b), *p* < 0.01). Results of EdU and clone formation experiments showed that the numbers of EdU staining positive cells and clone cells in the sh-circDENND4C group decreased compared with those in the blank control group (Figures 2(c) and 2(d)). The number of invasive cells in the sh-circDENND4C group was reduced (Figure 2(e)). After circDENND4C was silenced, the expression levels of EMT markers Vimentin, Twist1, and Snail1 also decreased (Figures 2(f)–2(h)).

### 3.3. Silencing circDENND4C Inhibits Lung Cancer Tumor Proliferation and Metastasis

**Figure 3:**
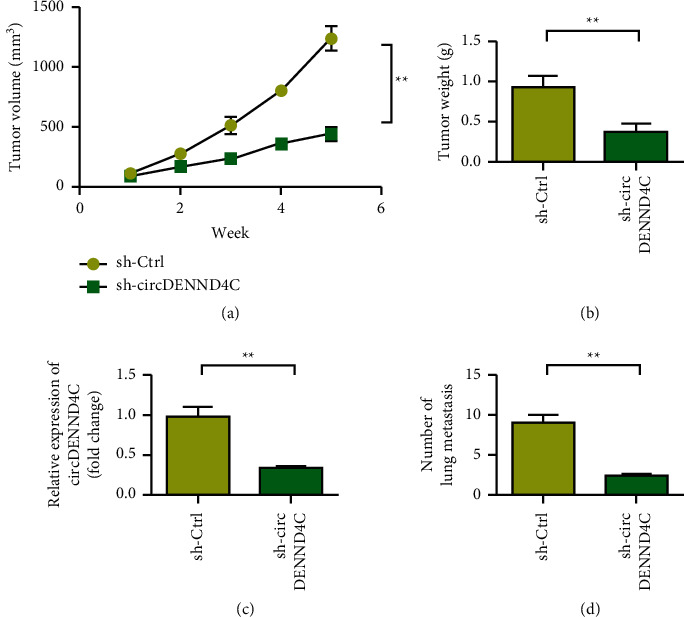
Downregulation of circDENND4C inhibits the proliferation and invasion of NSCLC in vivo. (a) sh-Ctrl and sh-circDENND4C subcutaneous transplanted tumor volume. (b) Tumor weight change curve. (c) PCR to detect the relative RNA expression of circDENND4C in subcutaneous tumors. (d) Statistics on the number of lung metastases. ^∗^*P* < 0.05 and ^∗∗^*P* < 0.01.

Tumor growth was observed during the subcutaneous tumor-bearing period. From the second week, tumors in the sh-circDENND4C group began to show growth inhibition. At the end of observation on day 35, the sh-circDENND4C group had inhibited lung cancer tumor growth, and the mean tumor volume and mass in the sh-circDENND4C group decreased (Figures 3(a) and 3(b)). qRT-PCR results showed that the expression level of circDENND4C in the sh-circDENND4C group was significantly lower than that in the blank control group (Figure 3(c), *p* < 0.01). Compared with the control group, the number of lung tissue metastases in the sh-circDENND4C group was significantly reduced (Figure 3(d), *p* < 0.01).

### 3.4. circDENND4C Can Target and Regulate the Expression of miR-141-3p

**Figure 4:**
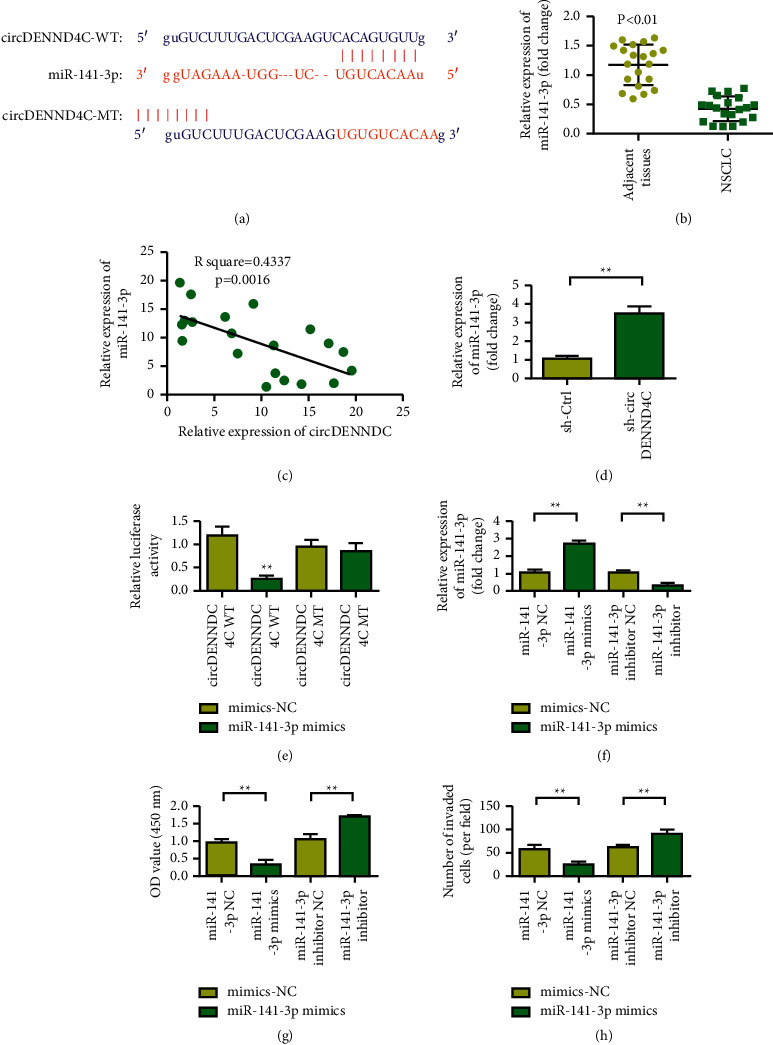
circDENND4C acts as a sponge to adsorb miR-141-3p, and miR-141-3p inhibits the proliferation and invasion of NSCLC. (a) miR-141-3p is determined as the target of circDENND4C through StarBase website analysis. (b) RT-qPCR analysis of the expression of miR-141-3p in NSCLC tissues. (c) Pearson correlation analysis was used to study the correlation between circDENND4C and miR-141-3p. (d) The expression of miR-141-3p in sh-Ctrl and sh-circDENND4C groups. (e) The luciferase method was used to study the direct interaction between circDENND4C and miR-141-3p. (f) Detection of transfection efficiency of miR-141-3p mimics and miR-141-3p inhibitor. (g) Verifying the effect of miR-141-3p mimic or inhibitor on the proliferation of A549 and H1299 cells through functional experiments. (h) Functional experiments verify the effect of miR-141-3p mimic or miR-141-3p inhibitor on the migration and invasion of A549 and H1299. ^∗∗^*P* < 0.01.

Figure 4(a) shows the binding site between circDENND4C and miR-141-3p. The miR-141-3p expression level in lung cancer tissues was significantly reduced compared with that in normal adjacent lung tissues (Figure 4(b), *p* < 0.01). In lung cancer tumor tissues, circDENND4C showed negative coexpression correlation with miR-141-3p (Figure 4(c)). miR-141-3p in lung cancer cells in the sh-circDENND4C group was increased compared with that in the sh-Ctrl group (Figure 4(d)). The luciferase reporter gene assay results showed that, after transfection with WT-circDENND4C gene expression vector, the luciferase activity of WT-circDENND4C lung cancer cells in the miR-141-3p group was significantly decreased compared with that in the miR-Con group (*p* < 0.05). However, after transfection with mut-circDENND4C gene expression vector, no significant difference was observed in the luciferase activity of mut-circDENND4C lung cancer cells in the miR-141-3p group compared with that in the miR-con group (Figure 4(e)). The activity of lung cancer cells in the miR-141-3p mimics group was reduced. The cell activity of miR-141-3p inhibitor group was increased (Figures 4(f) and 4(g), *p* < 0.01). Transwell assay results showed that the invasion ability of lung cancer cells in the miR-141-3p mimics group was reduced compared with that in the miR-141-3p NC group. The miR-141-3p inhibitor group showed enhanced cell invasion ability (Figure 4(h)). Therefore, miR-141-3p inhibits the proliferation and invasion of lung cancer A549 cells.

### 3.5. miR-141-3p Can Regulate the Expression of BRD4 in a Targeted Manner

**Figure 5:**
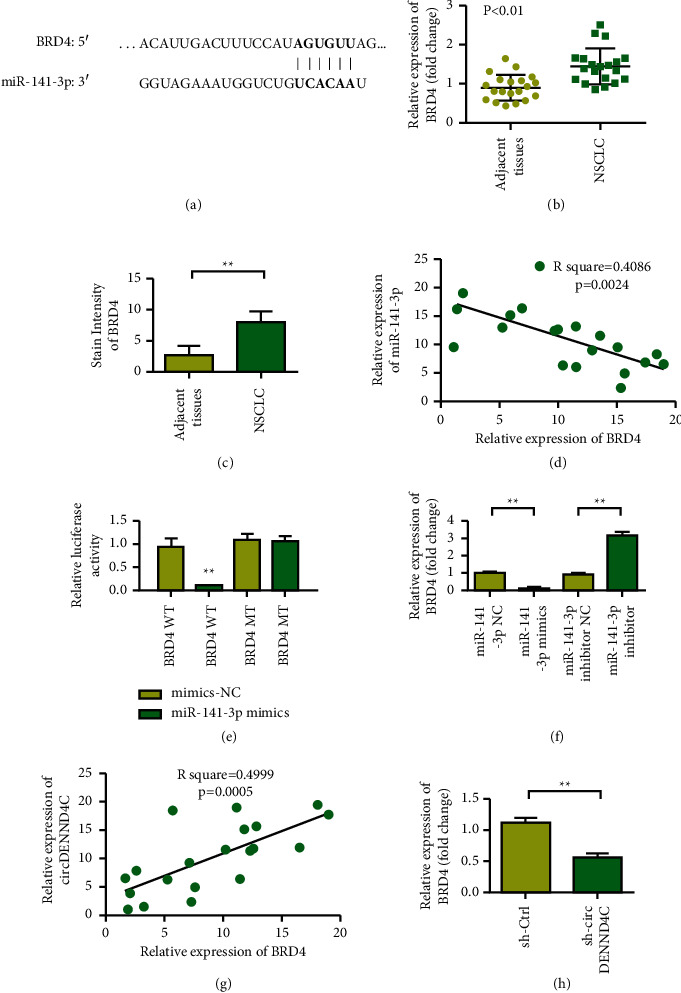
circDENND4C promotes cell growth and invasion by targeting the miR-141-3p/BRD4 axis. (a) The BRD4 gene is determined as a potential target gene of miR-141-3p by TargetScan analysis. (b) qRT-PCR detects the relative expression of BRD4 in the collected NSCLC tissues. (c) Immunohistochemical test further confirms the expression of BRD4 in NSCLC specimens. (d) Pearson correlation analysis of the correlation between BRD4 and miR-141-3p. (e) Luciferase method to study the direct interaction between BRD4 and miR-141-3p. (f) After transfection of miR-141-3p mimic or inhibitor in NSCLC cells, the expression of BRD4 mRNA was detected by PCR. (g) Pearson correlation analysis of the correlation between BRD4 and circDENND4C. (h) PCR was used to detect BRD4 mRNA expression in sh-Ctrl group and sh-circDENND4C group. ^∗^*P* < 0.05 and ^∗∗^*P* < 0.01.

Figure 5(a) shows the binding site between BRD4 and miR-141-3p. BRD4 in lung cancer tissues was higher (Figures 5(b) and 5(c), *p* < 0.01). In lung cancer tumor tissues, BRD4 showed a negative coexpression correlation with miR-141-3p (Figure 5(d)). The results of double luciferase reporter assay showed that miR-141-3p had a targeting relationship with BRD4 (Figure 5(e)). qRT-PCR results showed that, compared with that of the NC group, the expression level of BRD4 increased after overexpression of miR-141-3p. After miR-141-3p was inhibited, the expression of BRD4 increased (Figure 5(f)). Therefore, miR-141-3p can regulate the expression of BRD4 in a targeted manner. In lung cancer tumor tissues, BRD4 and circDENND4C showed a coexpression positive correlation (Figure 5(g)). The expression level of BRD4 in the sh-circDENND4C group was reduced (Figure 5(h)).

### 3.6. Knockdown of BRD4 or Overexpression of miR-141-3p Reverses the Oncogenic Effect of circDENND4C

**Figure 6:**
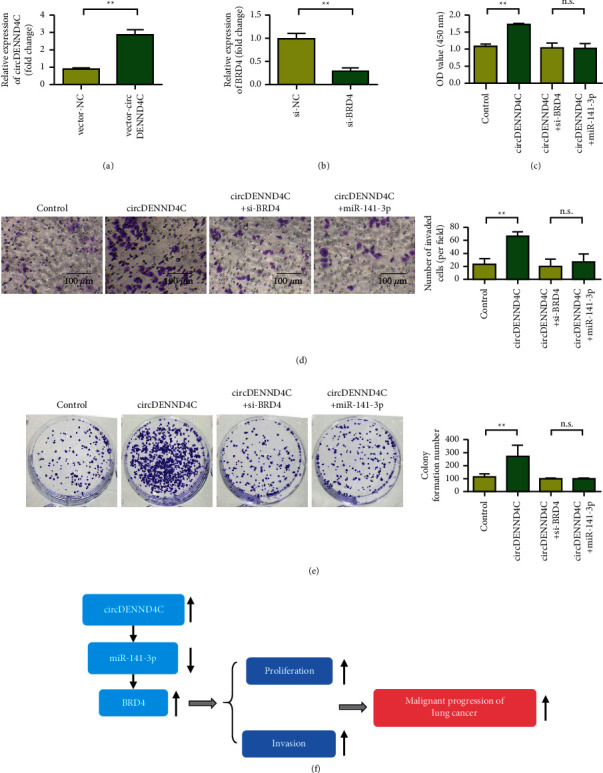
circDENND4C promotes the proliferation and metastasis of NSCLC cells through miR-141-3p/BRD4. (a) Detection of circDENND4C overexpression transfection efficiency. (b) Detection of transfection efficiency of siRNA BRD4. (c) The CCK-8 function recovery experiment was used to verify the cell proliferation ability of circDENND4C + si-BRD4 and circDENND4C + miR-141-3p group and circDENND4C group. (d) The Transwell function recovery experiment was used to verify the cell invasion ability of circDENND4C + si-BRD4 and circDENND4C + miR-141-3p group and circDENND4C group. (e) The clone formation function recovery experiment was used to verify the cell clone formation ability of circDENND4C + si-BRD4 and circDENND4C + miR-141-3p group and circDENND4C group. (f) The schematic diagram was used to clarify the potential molecular mechanism of circDENND4C through sponge adsorption of miR-141-3p, promoting the transfer and proliferation of NSCLC, and indirectly upregulating BRD4. ^∗^*P* < 0.05 and ^∗∗^*P* < 0.01.

The expression level of circDENND4C was higher in the vector-circDENND4C group (Figure 6(a)). After siRNA BRD4 was transfected into lung cancer cells, the expression of BRD4 was decreased (Figure 6(b)). CCK-8 detection results showed that the activity of lung cancer cells in the overexpressed circDENND4C group was significantly increased compared with that in the control group. Meanwhile, lung cancer cell activity was decreased after cotransfection of circDENND4C + si-BRD4 or circDENND4C + miR-141-3p at the same time (Figure 6(c)). Transwell and clonogenic assay results showed that the numbers of invasive cells and clone cells in the circDENND4C overexpression group were higher than those in the control group. In contrast, the numbers of invasive cells and cloned cells of lung cancer cells decreased after cotransfection of circDENND4C + si-BRD4 or circDENND4C + miR-141-3p (Figures 6(d) and 6(e)). Mechanism studies showed that circDENND4C, as an oncogene, upregulated the expression of BRD4 by inhibiting miR-141-3p, thus promoting the proliferation, invasion, and EMT process of NSCLC cells (Figure 6(f)).

## 4. Discussion

Compared with small cell carcinoma, NSCLC cancer cells grow and divide more slowly and spread and metastasize relatively late. NSCLC accounts for about 80%–85% of all lung cancers, and most patients are already in the middle and advanced stage when they are discovered, with a five-year survival rate below 54% [25]. One of the important causes of death in NSCLC is that its occurrence and development are a complex process that involves a variety of molecular mechanisms [26]. Studies have shown that circRNAs participate in the development of NSCLC [27].

circRNA plays its role by competing with miRNA to release downstream molecules, and it is involved in tumor genesis, proliferation, apoptosis, and invasion. The goal of tumor treatment is expected to be achieved by intervening in the circRNA level. Dai et al. [28] found that circ0006916 was downregulated in malignant transformation cells and lung cancer cells and tissues. It serves as a tumor suppressor in lung cancer by binding to miR-522-3p and inhibiting the activity of leucine-rich protein phosphatase 1 in the PH domain. Moreover, circ0006916 inhibits cell proliferation by slowing cell cycle progression. Zhu et al. [29] reported that Hsa_circ_0013958 was significantly upregulated in lung adenocarcinoma tissues, and the sponge identified as miR-134 could upregulate cyclin D1, which plays a key role in the occurrence and development of NSCLC. These results indicate that circRNA can participate in the occurrence and development of lung cancer and play a role in predicting the severity of lung cancer through its miRNA sponge function.

This study found that circDENND4C, as an oncogene, regulates the expression of its target gene BRD4 through its interaction with miR-141-3p, and it affects the proliferation, migration, invasion, and EMT processes of NSCLC cells. The results of this study showed that circDENND4C was highly expressed in NSCLC tissues. However, miR-141-3p had low expression in NSCLC tissues, and it targets the 3′UTR of BRD4 to inhibit proliferation and invasion of NSCLC cells. Downregulation of circDENND4C expression can inhibit proliferation and migration of NSCLC cells. Animal experiments showed that downregulation of circDENND4C could inhibit the proliferation and metastasis of tumor cells.

miRNA is widely involved in the occurrence and development of tumors and is closely related to the biological regulation of tumors [30]. Findings show that miR-141-3p was significantly overexpressed in chemotherapy-resistant epithelial ovarian cancer, participating in the chemical resistance process. Moreover, miR-141-3p is upregulated in prostate cancer and can promote prostate cancer cell proliferation by inhibiting KLF9 expression [31]. It also inhibits the growth and metastasis of osteosarcoma cells by targeting the epidermal growth factor receptor and influencing its downstream pathway proteins [32]. In this study, miR-141-3p in lung cancer tissues was reduced, thus showing an anticancer effect by targeting BRD4.

BRD4 has been reported to be able to promote the progression of gastric cancer by regulating c-MYC transcription and epigenetics [33]. Targeting the epigenetic signaling molecule BRD4 effectively inhibits cancer cachexia and prolongs survival [34]. In an in vitro experiment [35], the addition of BRD4 inhibitors showed a significant inhibitory effect on the growth and metastasis of colon cancer. In addition, BRD4 is upregulated in squamous cell carcinoma and can promote the growth and proliferation of squamous cell carcinoma cells [36]. When BRD4 expression is reduced, oral cancer cell growth and metastasis are inhibited [37]. In this study, upregulated expression of BRD4 in lung cancer was detected by RT-qPCR. TargetScan software was used to analyze the 3′UTR of BRD4 and miR-141-3p seed sequence, which were complementary to each other and are a potential target of miR-141-3p. To explore the relationship between BRD4 and miR-141-3p in lung cancer and its mechanism, luciferase assay was used to verify the targeting relationship between BRD4 and miR-141-3p. In addition, silencing BRD4 inhibits cell proliferation, migration, and invasion. Knockdown of BRD4 can reverse the oncogenic effect of circDENND4C.

## 5. Conclusion

This study showed that circDENND4C was highly expressed in lung cancer tissues and cells. Mechanism studies showed that circDENND4C upregulated the expression of BRD4 by adsorption of miR-141-3p as ceRNA, thus promoting the proliferation, migration, and invasion of NSCLC cells.

## Data Availability

All data included in this study are available upon request by contacting the corresponding author.
